# Tracking Changes in the Spring Barley Gene Pool in Poland during 120 Years of Breeding

**DOI:** 10.3390/ijms23094553

**Published:** 2022-04-20

**Authors:** Joanna Dziurdziak, Wiesław Podyma, Henryk Bujak, Maja Boczkowska

**Affiliations:** 1 Plant Breeding and Acclimatization Institute-National Research Institute, Radzików, 05-870 Błonie, Poland; j.dziurdziak@ihar.edu.pl (J.D.); w.podyma@ihar.edu.pl (W.P.); 2 Department of Genetics, Plant Breeding and Seed Production, Wrocław University of Environmental and Life Sciences, Grunwaldzki 24A, 53-363 Wrocław, Poland; henryk.bujak@upwr.edu.pl; 3 Research Center for Cultivar Testing (COBORU), 63-022 Słupia Wielka, Poland

**Keywords:** barley, DArTseq, SNP, diversity, cultivars, breeding

## Abstract

This study was undertaken to investigate the diversity and population structure of 83 spring barley (*Hordeum vulgare* L.) cultivars, which corresponded to 120 years of this crop’s breeding in Poland. The analysis was based on 11,655 DArTseq-derived SNPs evenly distributed across seven barley chromosomes. Five groups were assigned in the studied cultivars according to the period of their breeding. A decrease in observed heterozygosity within the groups was noted along with the progress in breeding, with a simultaneous increase in the inbreeding coefficient value. As a result of breeding, some of the unique allelic variation present in old cultivars was lost, but crosses with foreign materials also provided new alleles to the barley gene pool. It is important to mention that the above changes affected different chromosomes to varying degrees. The internal variability of the cultivars ranged from 0.011 to 0.236. Internal uniformity was lowest among the oldest cultivars, although some highly homogeneous ones were found among them. This is probably an effect of genetic drift or selection during their multiplications and regenerations in the period from breeding to the time of analysis. The population genetic structure of the studied group of cultivars appears to be quite complex. It was shown that their genetic makeup consists of as many as eleven distinct gene pools. The analysis also showed traces of directed selection on chromosomes 3H and 5H. Detailed data analysis confirmed the presence of duplicates for 11 cultivars. The performed research will allow both improvement of the management of barley genetic resources in the gene bank and the reuse of this rich and forgotten variability in breeding programs and research.

## 1. Introduction

The International Treaty on Plant Genetic Resources for Food and Agriculture (ITPGRFA) in 2001 defined cultivar as “a plant grouping, within a single botanical taxon of the lowest known rank, defined by the reproducible expression of its distinguishing and other genetic characteristics” [1].

The advances in plant breeding achieved in the 20th century have had a tremendous impact on the agricultural landscape and have contributed to improving global food security through significant increases in crop productivity [2,3]. A milestone was the Green Revolution of the 1960s and 1970s [4]. However, it is believed that this was a major trigger for the genetic erosion of crop species, and constant selection based on crosses between genetically closely related cultivars has significantly narrowed the crops’ gene pools [5,6].

Barley breeding has focused on improving yield and biotic and abiotic stress tolerance. Malting quality is also important due to barley’s use in the brewing industry. Various traditional breeding methods have been employed, i.e., selection (mass, pure line, pedigree or bulk), haploid and doubled haploid production, mutation, single-seed descent (SSD), compound crosses, backcrossing, interspecific and intergeneric crosses. Male sterile-facilitated recurrent selection (MSFRS) and diallel selective mating system (DSMS) have also been used, which facilitate breakage of existing linkage blocks and expansion of the gene pool by providing large amounts of genetic diversity into barley cultivars [7]. Due to the increasing dynamics of changes in market demands and needs, due to climate change and the emergence of new pathogen races, the most traditional methods, requiring a long-term breeding program, have needed support. Molecular biology and genetic engineering tools have provided a significant shortening of the breeding process [8,9]. Molecular markers, Quantitative Trait Loci (QTL) mapping and finally whole-genome sequencing, as well as genetic modification and genome editing have facilitated early generation and targeted selection and thus overcome the disadvantages of traditional breeding [8].

Using molecular biology tools in breeding has significantly facilitated and accelerated the identification of genotypes that determine a specific and desired phenotype. The molecular characterization of preserved collections performed in gene banks helps in the preliminary identification of germplasm potentially applicable to current breeding programs. This is crucial, especially where there is a fragmented structure of the breeding companies producing cultivars for the local market, which usually do not have the financial resources and laboratory infrastructure to perform their own rapid screening of gene bank collections to identify components for crossbreeding. However, it is essential to provide open access to genetic data.

The beginning of spring barley breeding on Polish territory dates back to the end of the 19th century. Beginning in 1870, breeding stations and companies were established in the partitioned Polish territory. Initially, breeding was dominated by cultivars selected from elite materials imported from abroad, landraces and ecotypes. Barley breeding in Wielkopolska, which at that time was part of the Prussian partitioning, was carried out by Hildebrand, Kirhoff and Stiegler. Their cultivars were widely grown on Polish lands and used in further breeding work. At the beginning of the 20th century, Polish breeders started to work on components and cultivars from Moravia (now the Czech Republic) [10]. As early as in 1902, Antoni Sempołowski, who is considered the pioneer of Polish breeding, distinguished four ways of cereal improvement, i.e., improvement by selection, breeding of new cultivars by searching and consolidation of new types, breeding of new cultivars by crossing, and acclimatization [11]. In the interwar period, barley breeders began crossing indigenous landraces with German cultivars, including the most valued cultivar, ‘Isaria’ [12]. In later periods well yielding, popular in the cultivation of foreign cultivars and Polish parental components, was used for further crossbreeding [10]. Old cultivars can be a valuable source of variability that has been lost due to the focus on high yield [13]. They may contain genes determining resistance to biotic and abiotic stresses, as well as parameters determining, quality oriented towards health-promoting properties [14,15,16]. Therefore, old cultivars and landraces are a source of genetic information for direct use or as parental lines in breeding programs for better adaptation of new cultivars [17,18,19]. However, it should also be considered that the general profile of agrotechnical traits will be significantly worse in the case of old cultivars compared to modern ones [20].

Here, emphasis was placed on investigating changes in the gene pool of the Polish spring barley cultivars collection during 120 years of breeding based on the analysis of DArTseq-derived SNPs. For five breeding periods, both the size of gene pools, their structure and internal level of diversity were assessed. Traces of targeted breeding were examined along the chromosomes. The level of heterogeneity within the studied cultivars was also estimated. The analysis presented here also provided an opportunity to verify and identify duplicates in the germplasm collection. The DArTseq results also enabled the core collection to be selected. Therefore, the results will improve the efficiency of collection management and its use in research and breeding.

## 2. Results

### 2.1. Data Quality Analysis

**Figure 1:**
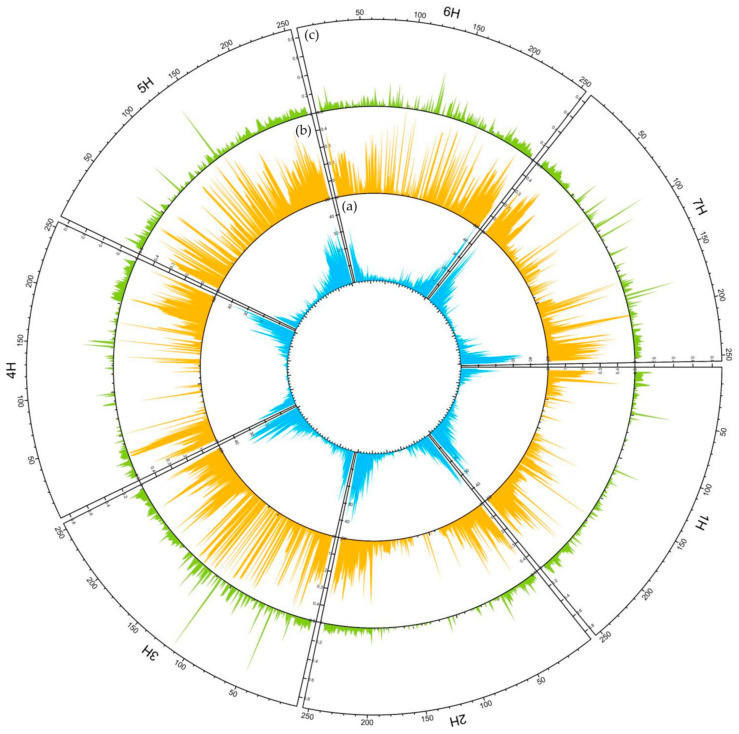
Circular overview of seven *H. vulgare* chromosomes based on DArTseq data acquired for 83 spring cultivars. (**a**) DArTseq loci distribution; (**b**) Average polymorphism information content (PIC) distribution; (**c**) Average observed heterozygosity (Ho) distribution. A sliding window approach with 500 kb windows, printed for 250 positions along the full length of barley chromosomes based on the genome assembly: IBSC_v2 [21] was applied.

Sequencing yielded over 75,000 SNP loci, from which loci with low reproducibility (RepAvg ≤ 0.95), low call rate (CallRate ≤ 0.95), and low minor allele frequency (MAF < 0.01) were removed. As a result, approximately 85% of the loci obtained were removed, and loci, 11,655 in number, that met all quality parameters were used for analysis. The distribution of loci on chromosomes before and after filtering was also checked. Filtering did not affect the uniform distribution on chromosomes, which ranged from 10 to 15%. However, the proportion of loci with unknown chromosomal location decreased by 2% compared to the raw data. The highest number of loci analyzed was located on chromosome 2H (1695) and the lowest on 1H (1150). On average, 1 bp per 470 Kbp in the genome was analyzed. Loci distribution along each chromosome showed a similar pattern, i.e., the number of studied loci was higher at the ends of chromosomes and decreased towards centromere (Table 1, Figure 1).

The frequencies of transitions (A > G, G > A, C > T, and T > C) and transversions (other variants) among SNPs were 60.2% and 39.8%, respectively. On chromosome 3H there were significantly less purine transitions and significantly more pyrimidine transitions compared to the other chromosomes. Among the transversions, their significantly decreased frequency was observed on 1H (A > C) and 3H (A < T), and a simultaneously increased frequency on 7H (C > A) (Table 2).

**Figure 2:**
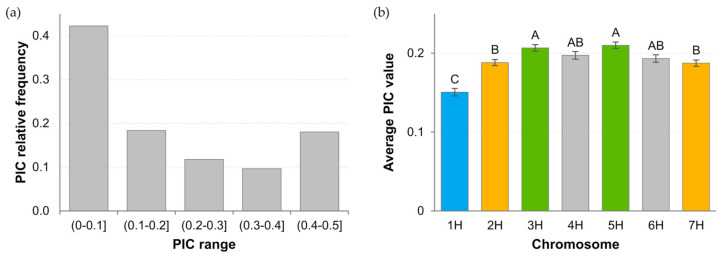
Summary of polymorphism information content (PIC) values. (**a**) Range of relative frequencies for all analyzed DArTseq loci in 83 spring barley cultivars; (**b**) Mean PIC value including chromosomal location of studied DArTseq loci. Letters above the bars in the graph indicate homogeneous groups determined by Tukey’s post hoc test.

The polymorphism information content (PIC) ranged from 0.02 to 0.49 with mean 0.19 and median 0.13. Over 40% of loci had PIC below 0.1 (Figure 2b). Between 34% (5H) and 52% (1H) of low PIC loci were present on individual chromosomes. In total, about 18% of loci were highly informative, i.e., had a PIC above 0.4. Their proportion on chromosomes ranged from 0.12 (1H) to 0.22 (3H). The mean PIC value for each chromosome showed significant. differences. The lowest value was observed for 1H and the highest values for 3H and 5H (Figure 2b).

### 2.2. Genetic Diversity

**Figure 3:**
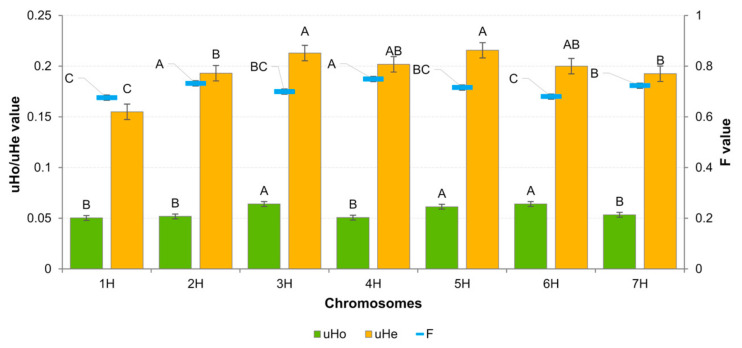
Summary of the diversity coefficient values across barley chromosomes for 83 cultivars based on DArTseq data. Letters above the bars in the graph indicate homogeneous groups determined by Tukey’s post hoc test.

The coefficients of variation such as observed heterozygosity (uHo), expected heterozygosity (uHe) and fixation factor (F) were calculated for the studied material. The mean values of these were 0.058, 0.197 and 0.706, respectively. The mean uHo for 3H, 5H and 6H (~0.06) was significantly higher than for the other chromosomes (~0.05) (Figure 3). The mean uHe was 0.155–0.216 for 1H and 5H, respectively. F-values for individual chromosomes also showed significant differences. The lowest value was observed for 1H (0.671) and the highest for 4H (0.757). Heterozygous state was not observed in approximately 23% of loci. Chromosome 5H had the highest proportion of heterozygous loci, while 2H had the lowest (Table 2).

**Figure 4:**
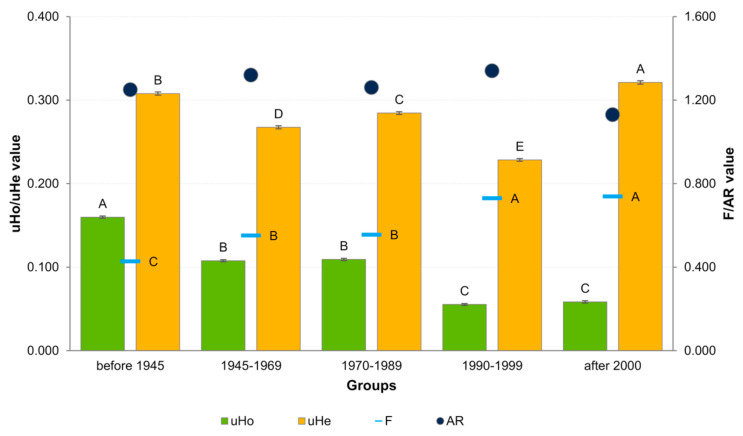
Summary of the diversity coefficient values for cultivar groups assigned based on the breeding date. Letters above the bars in the graph indicate homogeneous groups determined by Tukey’s post hoc test.

The analysis of diversity coefficients (uHo, uHe and F) in groups of cultivars, assigned based on the period of breeding, showed the presence of significant differences (Figure 4). Heterozygosity observed decreased progressively with time, while the level of inbreeding increased. The pattern of uHe values was a little more complicated, i.e., it tended to alternately decrease and increase in consecutive periods. Its values were highest in the group of the newest and the oldest cultivars and lowest in the group from the period 1990–1999. Allelic richness (AR) also showed fluctuations over time, being highest in the period 1990–1999 and lowest in cultivars bred after 2000.

**Figure 5:**
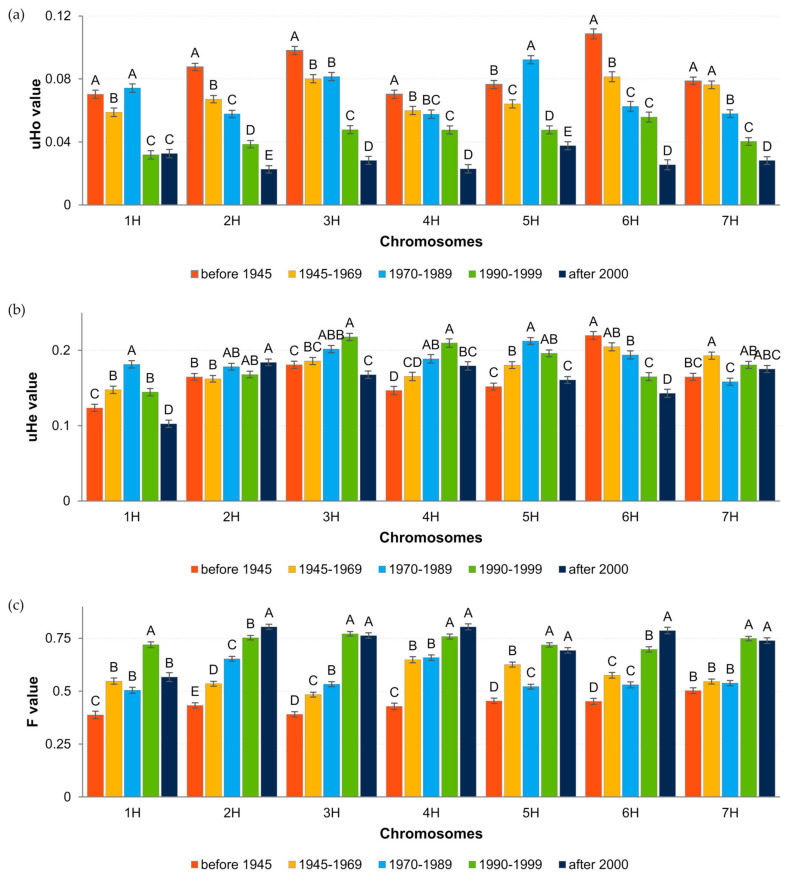
Summary of the diversity coefficients values for cultivar groups, assigned based on the breeding date considering chromosome localization. Letters above the bars in the graph indicate homogeneous groups determined by Tukey’s post hoc test. (**a**) observed heterozygosity (uHo); (**b**) expected heterozygosity (uHe); (**c**) inbreeding coefficient (F).

Analysis of the diversity coefficients in relation to the period of breeding and chromosome showed that the pattern of changes in the level of uHo was in most cases consistent with the main pattern (Figure 5). The divergence occurred on chromosomes 1H and 5H, where an increase in heterogeneity of cultivars bred in 1970–1989 was observed. uHo showed a variable behavior over time depending on the chromosome. For 1H, 3H, 4H and 5H uHe initially increased and then decreased, although the increasing trend interruption occurred either in the period 1970–1989 or in 1990–1999. For 6H uHe decreased with time and for 2H and 7H it fluctuated. The inbreeding level showed a similar change pattern as uHo, but in the opposite direction.

**Figure 6:**
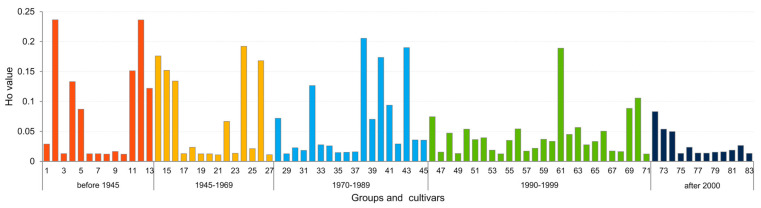
Heterogeneity level of 83 spring barley cultivars expressed by observed heterozygosity value based on SNPs derived from DArTseq analysis.

Because DArTseq analysis was conducted on pooled samples, where each cultivar was represented by eight seedlings, it was possible to estimate the level of intrinsic heterogeneity of the cultivars studied. Barley is a self-pollinating species; therefore, heterozygous loci are due to the presence of different genotypes in the sample. Thus, it can be assumed that the heterozygosity observed reflects the heterogeneity of the cultivar. Within 83 tested cultivars Ho ranged from 0.011 (‘Kazimierski’) to 0.236 (‘Cesarski Stieglera’) (Figure 6). In the group of the oldest cultivars, i.e., those bred before 1945, which included also cultivars bred at the end of the 19th century, the level of heterogeneity ranged from 0.012 (‘Przeworski’) to 0.236 (‘Cesarski Stieglera’). Eight cultivars showed a relatively high level of homogeneity, while the remaining five were significantly internally differentiated. In cultivars bred between 1945 and 1969, heterogeneity ranged from 0.011 (‘Kazimierski’) to 0.176 (‘Antoniński Browarny’). In cultivars bred between 1945 and 1969, heterogeneity ranged from 0.011 (‘Kazimierski’) to 0.176 (‘Antoniński Browarny’). This group included two pairs of accessions whose passport data indicate that they may be duplicates. According to the EGISET database, accessions numbered PL42124 and PL43614 are duplicates of ‘Damazy’ cultivar and PL40940 and PL42363 of ‘Jarek’ cultivar. These accessions are characterized by a high level of homogeneity, although in the case of ‘Damazy’, there is a difference between the two samples, i.e., 0.013 vs. 0.024. Among 18 accessions representing cultivars bred in the period 1970–1989, only four showed an increased level of heterogeneity (>0.1), i.e., ‘Lot’ (0.205), ‘Polon’ (0.190), ‘Lubuski’ (0.174) and ‘Dema’ (0.127). In this group, there were as many as six pairs of accessions that may represent duplicates (Table 1). For two pairs, i.e., PL43033 and PL43416 (‘Dema’) and PL43032 and PL43421 (‘Lot’), there were significant differences in the level of heterogeneity. In the fourth and most numerous group of cultivars, which were bred in the late 20th century, the level of heterogeneity was quite even and noticeably low (generally below 0.09). Accession number PL43812 is an exception; according to passport data, it is one of three accessions representing the ‘Bryl’ cultivar. However, the level of heterogeneity of this accession (0.189) is considerably higher than that of the other two accessions, for which Ho is about 0.035. A value above 0.1 in this group was also found in the sample representing the ‘Rataj’ cultivar. The fifth group consisted of modern cultivars, among which there were seven of Polish origin and five of foreign origin, i.e., from Germany and France. All cultivars were characterized by a very high level of homogeneity. The highest Ho value was found in the ‘Granal’ cultivar (0.083), the lowest in ‘Runner’ and ‘RGT Planet’ (0.013).

### 2.3. Unique Alleles

**Figure 7:**
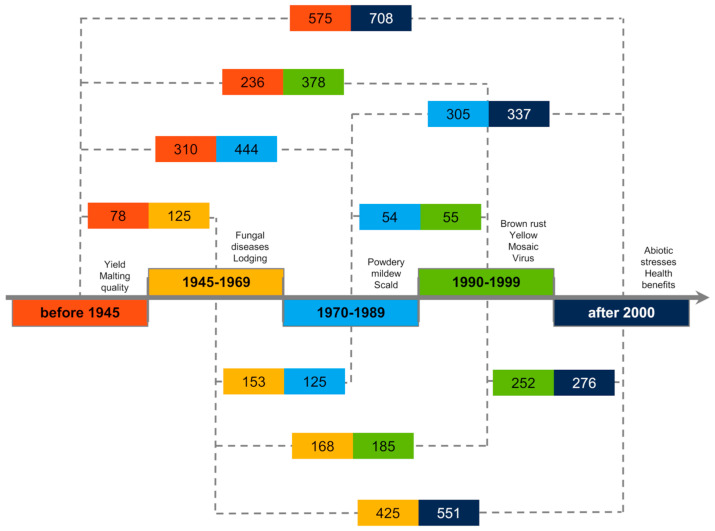
Summary of changes in the number of unique alleles during more than 120 years of breeding and cultivation of spring barley in Poland. Colors indicate groups and dashed lines connect compared periods. Above the axis, information about the new breeding objectives is placed.

The number of unique alleles was also compared among the groups (Figure 7). As a threshold level, the frequency of a unique variant higher or equal to 0.25 in a given group of cultivars was assumed. In this way, the dynamics of changes in the genome of the presence of unique variants occurring quite commonly in the studied groups was observed. Data considering rare alleles, i.e., >0.05, are presented in Table S1. In the oldest cultivars, 78 loci contained variants that were not transferred to the group of cultivars bred in the subsequent period. However, in the group of cultivars bred between 1945 and 1969, there were 125 loci in which new variants were present. Thus, changes affected about 1.74% of all investigated loci. The highest proportion of changes of unique alleles was observed between the groups of cultivars bred in 1990–1999 and modern ones, and they were related to 4.53% of analyzed loci. On the other hand, the smallest changes were observed between the groups from the middle breeding period, i.e., between 1970–1989 and 1990–1999 (0.94% of loci). Changes in allele frequency, i.e., the disappearance of ‘old’ alleles and appearance of ‘new’ ones, are related to the constant evolution of the breeding direction and to the appearance of new objectives, apart from yield increase.

From the perspective of individual chromosomes, the greatest magnitude of change was in chromosome 5H (Table 3). During the surveyed breeding period, 125 unique allelic variants were lost while 138 new variants were introduced. The smallest changes affected 1H and 6H; however, on 1H almost twice as many new allelic variants appeared as were lost, while on 6H only the removal of variation associated with unique alleles took place. Comparing the different consecutive periods, it is clear that the dynamics of change varied at different times for different chromosomes. However, two points at which “old” variation was replaced by “new” variation can be clearly seen, i.e., 1970–1989 and recently.

### 2.4. Genetic Distance and Principal Coordinate Analysis

An analysis of genetic distance showed that the lowest distance occurred between the two accessions representing the “Klimek” cultivar, and the highest between ‘Mazowiecki’ and ‘Stratus’ (Table 4). Low distance values, i.e., below 0.05, were also observed for nine successive pairs of accessions. This similarity will be discussed in detail in the following section, dealing with duplicates. Maximal genetic distance between accessions in the five groups had the lowest value for modern cultivars, and the highest for cultivars bred in the period 1945–1969. Thus, it can be concluded that, among the studied groups of cultivars, those bred most recently have the narrowest gene pool, while the widest gene pool was recorded for cultivars bred after World War II.

**Figure 8:**
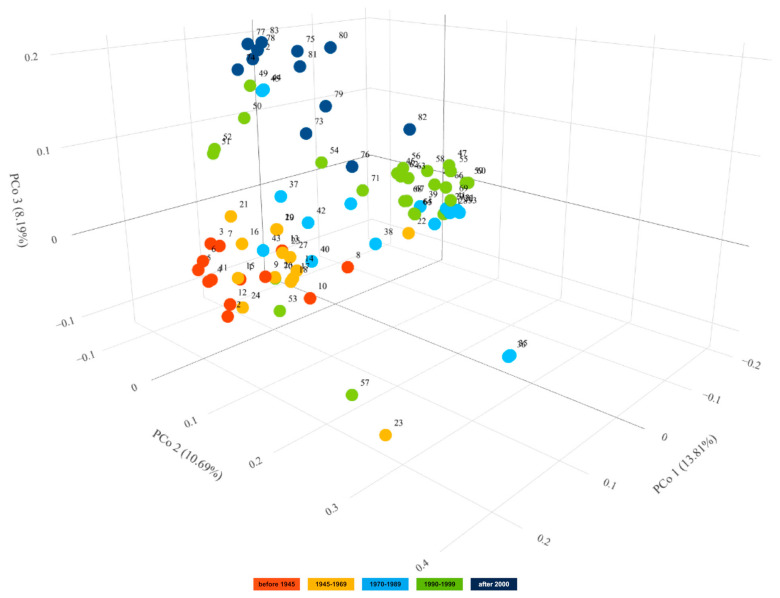
Graphical presentation of the Principal Coordinate Analysis results for DArTseq data of 83 spring barley cultivars. Results in the first three coordinates’ system. Each point denotes one tested cultivar. Numbering according to Table 6. Rotable 3D figure can be found in the supplementary materials (Figure S1).

Principal coordinate analysis (PCoA) performed for 83 spring barley cultivars indicated that the first three axes account for 32.69%, i.e., 13.81%, 10.69% and 8.19% of the variation, respectively (Figure 8). Graphical visualization of the results in a 3D plot of the first three coordinates showed that cultivars bred in the first four periods were arranged sequentially along the PCo1 axis. There is no clear demarcation between the groups of cultivars, and the gene pools in the subsequent periods partly overlap and intermingle. The PCo3 axis allowed us to distinguish the group of the newest cultivars. Several cultivars bred in the period 1990–1999 (‘Orlik’_(51,52)_, ‘Mobek’ and ‘Gwarek’) exhibit greater similarity to the group of recent cultivars than to cultivars bred in the same period. Among the most recent cultivars, those bred in Poland display a link to historical domestic materials. Foreign cultivars, on the other hand, show some distinctness. Polish cultivar ‘Podarek’ is the most genetically similar to foreign cultivars, especially to ‘Alianz’ and ‘RGT Planet’. The 3D plot also clearly shows the distinctiveness of the five accessions. Among them, the outermost, i.e., ‘Klimek’_(35,36)_ and ‘Mazowiecki’, are multi-row. The other two are ‘Polo’ and ‘Start’. Both are two-row, like the rest of the tested cultivars, but they originated from crosses of foreign cultivars.

### 2.5. Population Structure

Analysis of Molecular Variance (AMOVA) performed for 83 spring barley cultivars assigned to five breeding periods showed that most of the variation occurred within the groups (91%), and only 9% was inter-group variation.

**Figure 9:**
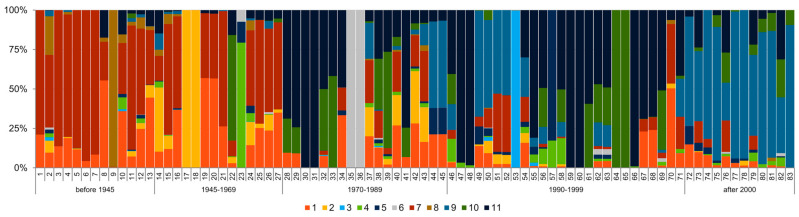
The results of 100,000 iterations of STRUCTURE software [22] for 83 spring barley cultivars based on DArTseq-derived SNPs data with K values K = 11 based on ad hoc measure ∆K [23,24], where K is the number of ad hoc clusters; each vertical bar represents one cultivar that is marked by order number according to Table 6. The length of the colored segment shows the estimated proportion of membership of each gene pool in the cultivar genetic makeup.

**Figure 10:**
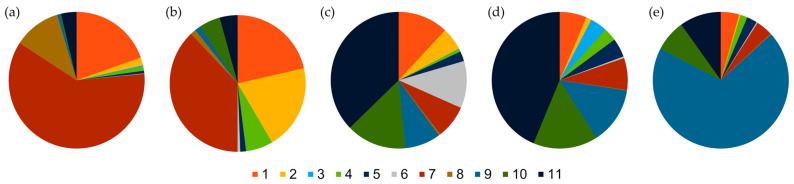
Proportion of 11 gene pools in five breeding periods of spring barley based on population structure analysis. (**a**) Cultivars bred before 1945; (**b**) cultivars bred between 1945 and 1969; (**c**) cultivars bred between 1970 and 1989; (**d**) cultivars bred between 1990 and 1999; (**e**) cultivars bred after 2000.

The admixture model in the STRUCTURE software [22] was implemented to investigate the population structure in the studied set of cultivars. Based on ad hoc statistic ΔK, the true number of clusters in the current study was identified at the level of 11 (Figure S2). Cultivars were assigned into clusters based on an 80% membership threshold. Only 28 cultivars were classified into nine clusters, i.e., gene pools, and the rest showed varying levels of admixture (Figure 9). Most cultivars were assigned to pools 11 (nine cultivars) and 9 (seven cultivars). None of the studied accessions were assigned to pools 1 and 4 (Figure 10). The group of cultivars bred before 1945 was dominated by pool 7, as in the following period (Figure 10). However, it should be noted that the percentage of this cluster decreased from 60.7% to 37.9% in the following periods. What is more, in the group of the oldest cultivars, five were considered pure, i.e., four were assigned to cluster 7 and one to cluster 8. The share of cluster 8 in the later periods of breeding is negligible and practically does not occur in cultivars bred after 1969. About 20% of this group was also represented by cluster 1. Its highest admixture was observed in the cultivar ‘Kujawski’. The participation of the remaining gene pools did not exceed several percent. In the group of cultivars bred in the period 1945–1969, only two cultivars were recognized as belonging to cluster 2. In both cases, this was ‘Damazy’. It is worth noticing that this cluster appears as an admixture in several more cultivars, but its contribution does not exceed 40%. In this group, the proportion of gene pool 1 increases slightly (21.4%). This pool constitutes about 57% of the genetic makeup in the cultivar ‘Jarek’, represented by two accessions. In the remaining cultivars, its content ranged from 0 to 37%. In the next two periods, in total, from 1970 to 1999, pool 11 was dominant and its participation increased with time from 37.3% to 43.7%. Among the cultivars bred in the initial period (1970–1989), only four were classified as pure. Two accessions representing ‘Klimek’ cultivar were assigned to gene pool 6, and two representing ‘Bielik’ cultivar to pool 11. Interestingly, gene pool 6 was practically absent in the remaining cultivars. In the group of cultivars from a later period (1990–1999), 10 cultivars were considered pure. They represent cluster 11 (6 accessions), 10 (two accessions) and 3 and 5 (one accession each). At the same time, the share of cluster 1 decreased with time in these two groups. The proportion of cluster 10 remained constant at about 15%, while an increase from 8.5% to 13.5% was observed for cluster 9 in these two groups. Whereas for the previous four periods of breeding, continuity of changes in population structure was observed, in the group of contemporary cultivars there was a rapid increase in the contribution of cluster 9, to 69.4%, and marginalization of other clusters. Seven cultivars from this period were assigned as pure to cluster 9, while in the rest its participation ranged from 23.8–69.4%. It should be noted that five cultivars with the highest proportion of cluster 9 were of foreign origin. An additional contribution of clusters 10 and 11 was observed in Polish cultivars. An exception was ‘Podarek’ cultivar, whose genetic makeup does not differ significantly from Western European cultivars.

### 2.6. Traces of Targeted Selection

**Figure 11:**
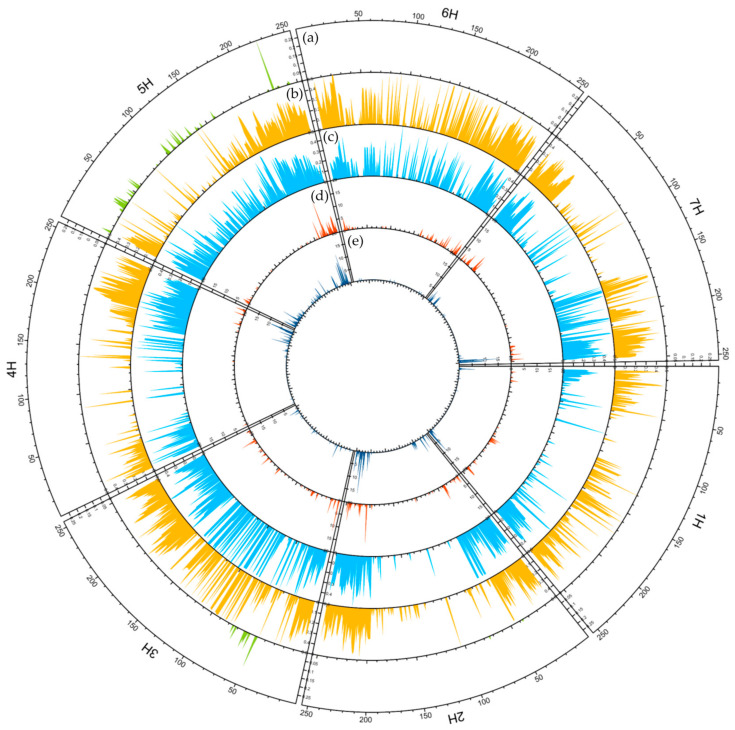
Circular overview of seven *H. vulgare* chromosomes. (**a**) Transformed FST^10^ for cultivars bred before 1945 and after 2000; (**b**) Average polymorphism information content (PIC) distribution in cultivars bred before 1945; (**c**) Average polymorphism information content (PIC) Distribution in cultivars bred after 2000; (**d**) Number of unique SNPs in cultivars bred before 1945; (**e**) Number of unique SNPs in cultivars bred after 2000. A sliding window approach with 500 kb windows, printed for 250 positions along the full length of barley chromosomes based on the genome assembly: IBSC_v2 [21].

Genomic regions involved in differentiation of cultivars bred before 1945 and after 2000 were revealed by plotting FST values for all loci with known locations in the genome (Figure 11). Regions with high FST values that indicate fixation of different alleles in both groups were observed on chromosomes 5H and 3H. The majority of regions with high FST were identified on 5H. It is noteworthy that these regions were found in both distal (especially in the short arm) and pericentromeric parts. On 3H, high FST was observed on the short arm and these loci were located in the middle part of the arm. For comparison, the analysis of PIC distribution in the two groups of cultivars was also performed. It showed that in the majority the PIC profile remained unchanged. Importantly, regions of low polymorphism were found in centromeric and pericentromeric regions in both groups at 1H, 2H, 4H and 7H. A remarkable change in the PIC profile was detected at 5H; in the region with high FST, the average PIC value increased in the group of the most recent cultivars. The alleles not present in the oldest cultivars also appeared there.

### 2.7. Identification and Verification of Duplicates

**Figure 12:**
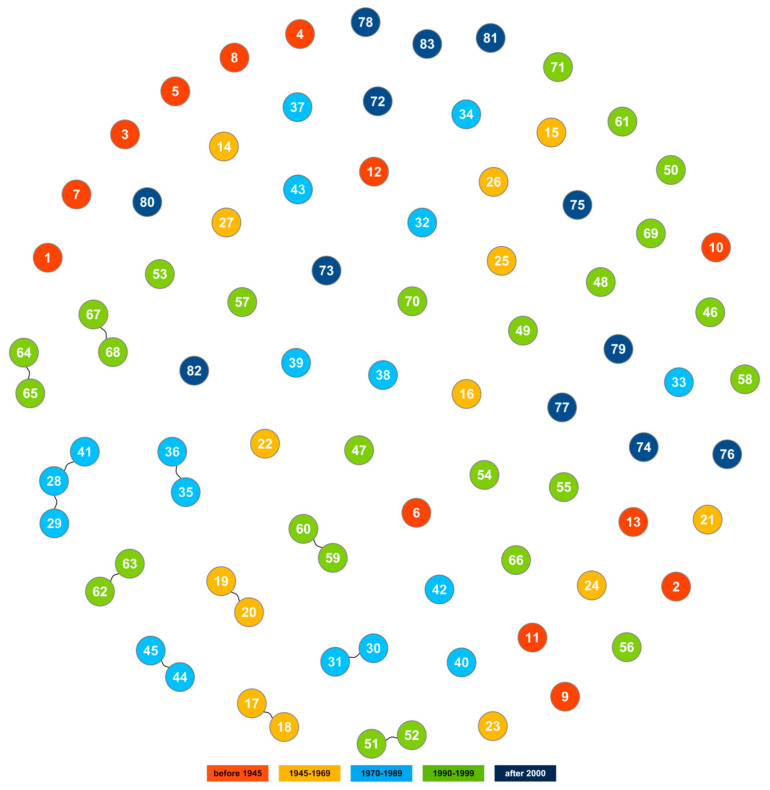
Identity by descent (IBD) based clustering of spring barley cultivars with cutoff at 0.95. Accession numbers according to Table 6.

In the studied set of 83 spring barley cultivars, as many as 31 accessions had passport data indicating that they appeared to be duplicates or even triplicates of cultivars. These accessions were submitted to the gene bank in different years. One of the aims of this study was to verify whether these accessions were indeed duplicates. For final verification, an identity by descent (IBD) analysis was performed (Figure 12) and its results were compared with those obtained in previously described analyses (Table 5). In this way, using different analytical approaches, it was possible to determine that duplicates occur for 10 varieties in the gene bank collection. An additional triplicate was identified for the cultivar Ars. The cultivar Mago showed a very high level of genetic similarity to both accessions of ‘Ars’ cultivar. However, in the case of three ‘Bryl ‘accessions, DArTseq analysis revealed genetic distinctness of accession PL 43812. It should therefore be assumed that this accession does not represent the ‘Bryl’ cultivar because the seed sample was contaminated with another cultivar, as indicated by its exceptionally high heterogeneity. Accessions representing cultivars such as ‘Dema’, ‘Lot’, ’Polo’ and ‘Rhodes’ according to passport data cannot be considered duplicates. Especially in the case of ‘Polo’, we are dealing with completely different genetic makeup. According to the population structure analysis, accession PL 43368 is the only one in the studied set of cultivars that represents the third gene pool and is therefore a valuable source of the collection diversity.

### 2.8. Core Collection

An advanced maximization strategy through a modified heuristic algorithm (A*), which is complete and optimal, i.e., it finds a path if only one exists, and the shortest path, was used to identify the minimum group of cultivars representing the full diversity. Out of the studied 83 cultivars, a set of 50 that should form the core collection was extracted. The cultivars are marked in Table 6.

## 3. Discussion

Described in this paper, the analysis of 83 spring barley cultivars representing 120 years of Polish breeding is the next step towards a molecular characterization of the collection conserved at NCPGR using high-resolution and genome-wide genotyping via the DArTseq method. This is a direct continuation of the study by Dziurdziak et al. [25,26] in which barley landraces were characterized. So far, a large number of articles have been published on the analysis of barley genetic diversity. In spite of this, the topic is still of interest to researchers from all over the world, which may indicate its relevance. In the last two years only, a number of publications on this subject have appeared [27,28,29,30,31,32,33,34,35]. A detailed description of genetic diversity is a prerequisite for effective conservation and utilization of genetic resources and progress in crop breeding programs.

### 3.1. SNP Abundance and Analysis of Base Changes

The analyzed loci, relatively uniform, represented all barley chromosomes, and their proportion and density was consistent with previous results obtained by DArTseq for barley [25]. At the same time, the analysis provided significantly more uniform and above 3.5 times denser data than the results obtained for wheat based on 65,560 loci derived from genotyping-by-sequencing (GBS), of which over 77% SNPs had unknown chromosome location [36].

The distribution of the analyzed loci along chromosomes, i.e., their high frequency in the distal parts of chromosomes and low or complete absence in the centromeric and pericentromeric regions, was also observed in previous studies on barley, durum wheat, and soybean [21,25,37,38]. This is also consistent with the distribution of protein-coding genes on barley chromosomes and the recombination rate [21]. A characteristic feature of Triticeae, including barley, is a significantly reduced level of meiotic recombination in the centromeric and pericentromeric regions [39,40,41]. A high recombination rate in distal chromosome fragments is associated with barley domestication. In wild barley, high recombination rates have been found in more interstitial chromosomes’ regions [42].

The analysis showed the presence of all possible SNP types in the studied cultivar set. The number of transition-type SNPs was 1.5 times higher than the transversion-type. An excess of transversions was also observed in previous studies involving NGS technology for cowpea, wheat, rice, barley, and common bean, among others [25,36,43,44,45]. The higher frequency of transition SNPs over transversion SNPs is due to their higher probability of preserving protein structure and function [44,46]. The most abundant SNP was A > G followed by C > T which may reflect the frequency of methylation/demethylation related mutations and was also common in the above cited studies. It is noteworthy that the DArT-seq analysis also revealed an increased relative abundance of C > G SNPs compared to the other transversions. Similar results were previously obtained by Duran et al. [47] for barley, Lai et al. [48] and Alipour et al. [36] for wheat, but this phenomenon has not been explained so far.

Polymorphism of the examined loci, determined by the PIC coefficient, was slightly lower in the cultivars than in the landraces previously studied [25]. However, differences occurred at the chromosome level. For landraces, the lowest mean PIC value was observed for 2H and for cultivars for 1H. This may indicate increased selection within 1H during breeding.

### 3.2. Genetic Diversity

For thousands of years, since their domestication, crops have been cultivated as populations with a complex genetic structure. Selection occurred on farms either as a result of human efforts or as a result of pressure from local ecogeographic conditions. This resulted in a differentiation between populations and the formation of landraces [49,50]. The 20th century brought progress in breeding and the displacement of landraces by cultivars tending towards homogeneity. To be released, cultivars had to go through evaluation for distinctness, uniformity, and stability [51].

Looking ove4 120 years of barley breeding in Poland it is clearly visible that the average variability within old cultivars is almost three times higher than in the group of modern cultivars, which are very uniform. Breeding-related selection is even more pronounced when the results obtained here are compared with the previous ones for landraces. Even the most internally differentiated cultivar, i.e., ‘Cesarski Sieglera’ (Ho = 0.236), is almost twice as less heterogeneous than the Polish landrace PL503844 (0.422) [25]. Thus, it can be clearly seen how breeding progress leads to genetic uniformity of individuals within a cultivar. Obviously, among the old cultivars studied here, there were also some with low heterogeneity, comparable even to modern cultivars, e.g., ‘Danubia Ciolkowski’ or ‘Kujawski’. However, it should be considered that a time lapse took place from the breeding of the oldest cultivars to their acquisition by the gene bank and finally to the time of the genetic analysis presented here. The oldest cultivars in the studied set come from the turn of the 19th and 20th century. Thus, they must have survived one or sometimes two world wars, during which part of their original variability may have been lost. Before these cultivars were acquired for the gene bank, they were maintained in the collections of breeders, universities or scientific institutes. Improper conservation breeding, repeated propagation or even lack of sufficiently frequent seed regeneration may have led to the degeneration of cultivars by further loss of variability. The breeders’ habit is to remove individuals diverging from the remaining plants from the cultivar, so that the cultivar fulfils the condition of uniformity. However, in the case of old cultivars, this may have exacerbated the loss of genetic variation. In the period prior to preservation in the seed bank, situations could also arise in which an old cultivar was deliberately over-selected for use in a breeding program, but this information was not provided to the gene bank. The low heterogeneity of some old cultivars may also be the result of genetic drift that occurred during seed reproduction for the gene bank, i.e., when the initial seed sample was too small and did not fully represent the original variability of the cultivar. Of course, at each of the stages the selection pressure of the environment may have also acted to remove some of the genotypes from the population, thus depleting its gene pool. At this point, from the point of view of the gene bank, it is irrelevant either where or for what reason the reduction in variation occurred. However, the information about the low level of heterogeneity attached to the description of the accessions in the gene bank database is important mainly for the end users, i.e., breeders and scientists, and sometimes also for farmers. Therefore, it cannot be generalized that old cultivars are always highly heterogeneous. It is worth noticing, that among old oat cultivars stored in NCPGR, and coming from the same breeding period, not so significant differences in the level of heterogeneity were observed [52]. However, the same trend was observed, i.e., that as breeding progressed in the 20th century, the genetic uniformity of individuals within a cultivar clearly increased [53]. However, the increase in genetic uniformity of the studied cultivars was not accompanied by a decrease in overall genetic diversity. Over the 120 years of breeding, fluctuations in the level of uHe, AR and maximum genetic distance were observed in the studied cultivar groups. Thus, no loss of genetic variation was observed as a result of breeding progress, as was implied by Gepts et al. [5] or Russell et al. [54]. The results of the analyses presented here are consistent with the meta-analysis of changes in genetic variation in crop cultivars conducted by van der Wouw et al. [55].

Based on the results obtained, no loss of genetic diversity was observed between the oldest and the newest cultivars studied. However, a detailed analysis of changes in allele frequency clearly indicated genetic erosion. In the course of breeding, about 600 alleles were lost from the gene pool of barley cultivars over the years. They have been preserved only thanks to the activity of the gene bank. Gradually, during breeding, ’old’ unique alleles were driven out from Polish cultivars and replaced by new allelic variation. As many as 11% of the 11,655 loci examined have completely different alleles in the group of the oldest and the newest cultivars. On the basis of the few pedigree data, we can state that alleles representing the native gene pool from landraces occurring in Poland and the Czech Republic were almost completely suppressed in breeding programs. This result also indicates that researchers should be very cautious about the results of the analysis of genetic diversity in the context of changes over time.

### 3.3. Evidence of Targeted Selection

Genome-wide DArTseq analysis provided an opportunity to evaluate changes in the genetic structure of spring barley cultivars bred in Poland. Both PCoA and STRUCTURE showed the merging of consecutive groups of gene pools. Breeding in Poland follows European trends, so it may be assumed that changes in population structure reflect a breeding focus on increasing yield and, in recent years, also on increasing resistance to pathogens. A gradient of variation and gradual targeted shifts were also observed in earlier studies on barley [56,57,58].

Thanks to the knowledge of the barley genome sequence and the mapping of DArTseq data to it, it was possible to determine the chromosomal localization of the analyzed loci. This allowed observation not only of the changes in genetic diversity in time, but also to what extent this affected individual chromosomes. In general, for most chromosomes there was the same pattern of change over time, i.e., a decrease in observed heterozygosity and an increase in inbreeding along with breeding progress. Comparison of the polymorphism level of loci along chromosomes in cultivars representing extreme breeding periods allowed detection of regions showing a lack of variation. These regions did not change during 120 years of breeding and were located in the centromeric and pericentromeric fragments of chromosomes 1H, 2H, 4H and 7H. Interestingly, in landraces of spring barley, such “empty” regions were observed at 1H, 2H and 4H [25] and, in the study of Tondelli et al. [58], at 1H, 2H and 7H. This means that landraces contain variability within 7H, and European modern cultivars within 4H, which is not present in Polish cultivars. The 4H centromeric region contains the QTL of net form net blotch (NFNB) resistance and *Mlg*, a powdery mildew resistance gene in the gene-dense pericentromeric region [59,60], while the 7H centromeric region contains QTLs related to heading date, yield and yield-forming traits such as plant height and root length [61,62,63,64,65,66].

FST analysis enabled identification of regions in which, during breeding, different alleles were fixed compared to the oldest cultivars. These regions occurred mainly on 5H. Their presence in the pericentromeric region was also found in modern European cultivars [58]. The fixation of “new” alleles in the pericentromeric region may be related to resistance breeding programs. In this region, several loci for resistance to leaf rust were found, including *Rph2* [58,67]. The VRN-1 gene encoding the MADS-box transcription factor is located in close proximity to the high-fixation region found on 5HL. Its involvement in the regulation of genes related to reproductive organs and flowering of plants is well known [68]. Wild-type VRN-1 determines the need for vernalization, i.e., prolonged exposure to cold as a prerequisite for flowering in most winter cereals [69]. Deletion in the first intron allows spring-sown plants to flower without prior vernalization [70]. It was proved that a genetic variation of VRN-1 correlates with flowering time in spring forms of barley [71]. According to the Voss-Fels et al. [72] study, VRN-1 is also associated with root system morphology. In addition, variation in this gene also affects final biomass and yield, especially under drought and salinity stress [73,74]. The high FST region on 3H may be associated with selection for reduced plant height and increased lodging resistance. Numerous genes and QTLs related to plant height have been mapped on chromosome 3H [58,75,76,77,78].

### 3.4. Improving the Management of the Germplasm Collection

DArTseq analysis will also enable improved management of the germ plasm collection. On the one hand, verification or identification of duplicate accessions was performed, and on the other, a core collection was selected. In a group of 74 cultivars stored in the Polish gene bank, for 15 cultivars, there were two or even three separate accessions.

Duplicates in gene banks arise when, by mistake, a cultivar or other type of accession becomes added to a collection multiple times [79]. Here, duplicates were most often created as a result of inclusion of accessions into the collection before their official registration as a cultivar and subsequent incorporation of an already registered cultivar. Accessions with identical passport data and genetic makeup will be combined as separate subsamples under a common accession number. In contrast, accession PL43812 ‘Bryl’ will have its passport data corrected. Due to the genetic distinctiveness of this accession from the other cultivars, it will be submitted to the curator for characterization and evaluation.

Improved barley collection management will also be provided by the selected core collection. The idea behind the establishment of core collections is to facilitate scientists and breeders in using the genetic resources stored in germplasm collections [80]. This also facilitates the maintenance of germplasm collections in gene banks, which can thus reduce the number of accessions held in active collections and provide access to the full range of diversity.

## 4. Materials and Methods

### 4.1. Plant Material

From the spring barley collection held at the National Center for Plant Genetic Resources (NCPGR), 74 accessions classified as advanced/improved cultivars were selected and analyzed. In addition, nine cultivars that are currently cultivated and have not yet been accessioned into the gene bank collection were included in the analysis (Table 6).

For each investigated cultivar, information about the period and place of its breeding and the time of its entry and presence in the official register of cultivated varieties, maintained by the Research Centre for Cultivar Testing (RCCT), was collected. Data for historical cultivars were obtained from Arseniuk et al. [10] and for more contemporary cultivars directly from RCCT. Based on these data, the cultivars were divided into five groups i.e., bred before 1945, 1945–1969, 1970–1989, 1990–1999 and after 2000.

**Table 6:** The list of spring barley cultivars analyzed by DArTseq.

No.	Accession Number	Cultivar Name	BREEDING SITE	Country	Year/Period of Registration	Year/Period of Deregistration	Core Collection
1	PL41572	Antoniński Browarny	Antoniny	POL	1930	1939	yes
2	PL41323	Cesarski Stieglera	Sobótka	POL	after 1918	1929	yes
3	PL42125	Danubia Ciołkowski (Danubia Ciślikowski)	Ciołkowo	POL	1930	1940	yes
4	PL40306	Elka Hilderanda	Kleszczewo	POL	1930	1939	no
5	PL41691	Hanna Borzymowicki	Borzymie	POL	1930	1939	yes
6	PL41692	Hanna Gambrinus; Hanna Gambrvnus	Sielce	POL	1918–1939	1957	yes
7	PL41695	Hanna Skrzeszowicki	Polanowice	POL	1918–1939	1971	yes
8	PL43217	Kujawski	Rusewko	POL	after 1918	1929	yes
9	PL41475	Kutnowski	Kutno	POL	1900	1918–1939	yes
10	PL42060	Puławski Browarny	Puławy	POL	1918–1939	1939	yes
11	PL41905	Przeworski	Dolne	POL	1918–1939	1939	yes
12	PL42129	Putza	Rusewko	POL	1930	1940	yes
13	PL40460	Teresa	Rusewko	POL	1930	1940	yes
14	PL41570	Antałek	Tulce	POL	1956	1971	yes
15	PL42034	Boryna	Szelejewo	POL	1955	1958	yes
16	PL42042	Browarny PZHR	Strzelce	POL	1946	1969	no
17	PL42124	Damazy	Polanowice	POL	1969	1975	yes
18	PL43614	Damazy	Polanowice	POL	1969	1975	no
19	PL40940	Jarek	Bąków	POL	1963	1970	no
20	PL42363	Jarek	Bąków	POL	1963	1970	no
21	PL41740	Kazimierski	Brzezie	POL	1955	1967	yes
22	PL44075	Kos	Leszno	POL	1946	1958	yes
23	PL42127	Mazowiecki	Młochów, Dłużew	POL	1946	1959	yes
24	PL41916	Refleks	Sobótka	POL	1955	1960	yes
25	PL41924	Sandomierski	Jasice	POL	1955	1960	no
26	PL41940	Skrzeszowicki	Polanowice	POL	1955	1972	no
27	PL41233	Wanda	Celbowo	POL	1965	1970	no
28	PL41419	Ars	Gorzów Wlkp.	POL	1983	1996	yes
29	PL43646	Ars	Gorzów Wlkp.	POL	1983	1996	no
30	PL43423	Bielik	Modzurów	POL	1984	1994	no
31	PL41415	Bielik	Modzurów	POL	1984	1994	yes
32	PL43033	Dema	Łagiewniki	POL	1987	1998	no
33	PL43416	Dema	Łagiewniki	POL	1987	1998	no
34	PL41328	Gryf	Gorzów Wlkp.	POL	1971	1980	no
35	PL43086	Klimek	Strzelce	POL	1989	1996	no
36	PL43414	Klimek	Strzelce	POL	1989	1996	yes
37	PL41329	Kosmos	Bąków	POL	1974	1978	yes
38	PL43032	Lot	Małyszyn, Gorzów Wlkp.	POL	1987	2007	yes
39	PL43421	Lot	Małyszyn, Gorzów Wlkp.	POL	1987	2007	no
40	PL41769	Lubuski	Strzelce, Borów	POL	1970	1975	no
41	PL41418	Mago	na	POL	na	na	no
42	PL41886	Piast	Polanowice	POL	na	1970	no
43	PL40556	Polon	Małyszyn	POL	1977	1989	yes
44	PL43056	Rudzik	Modzurów	POL	1987	2008	yes
45	PL43423	Rudzik	Modzurów	POL	1987	2008	no
46	PL44045	Atol	Strzelce	POL	1997	2007	no
47	PL44030	Bies	Modzurów	POL	1996	2010	yes
48	PL43637	Boss	Bąków	POL	1994	2020	yes
49	PL44031	Gwarek	Polanowice	POL	1999	2011	no
50	PL43424	Mobek	Modzurów	POL	1993	2001	yes
51	PL43335	Orlik	Bąków	POL	1990	2000	yes
52	PL43417	Orlik	Bąków	POL	1990	2000	no
53	PL43368	Polo	Strzelce	POL	1992	2003	yes
54	PL43411	Polo	Strzelce	POL	1992	2003	yes
55	PL43867	Rastik	Radzików	POL	1999	2010	yes
56	PL43749	Rodion	Radzików	POL	1996	2021	no
57	PL35393	Start	Polanowice	POL	1995	2010	yes
58	PL43868	Stratus	Strzelce	POL	1999	2020	yes
59	PL500074	Bryl	Bąków	POL	1998	2021	no
60	PL43949	Bryl	Bąków	POL	1998	2021	yes
61	PL43812	Bryl	Bąków	POL	1998	2021	no
62	PL500070	Edgar	Bąków	POL	1992	2004	no
63	PL500666	Edgar	Bąków	POL	1992	2004	no
64	PL43419	Nagrad	Nagradowice	POL	1990	na	no
65	PL43379	Nagrad	Nagradowice	POL	1990	na	yes
66	PL43750	Rabel	Radzików	POL	1996	2010	yes
67	PL500667	Rambo	Radzików	POL	1993	2003	yes
68	PL43747	Rambo	Radzików	POL	1993	2003	no
69	PL43748	Rataj	Radzików	POL	1996	2010	yes
70	PL43369	Rodos	Strzelce	POL	1992	2010	yes
71	PL43412	Rodos	Strzelce	POL	1992	2010	yes
72	PL503817	Granal	Nagradowice	POL	2001	2021	no
73	PL503818	Nadek	Nagradowice	POL	2004	2014	yes
74	PL44032	Sezam	Szelejewo, Modzurów	POL	2000	2010	no
75	ni	Runner	na	GER	2018	up now	no
76	ni	Atico	Kraków	POL	2009	up now	yes
77	ni	Podarek	Strzelce	POL	2014	up now	yes
78	ni	Allianz	na	FRA	2016	up now	no
79	ni	Rubaszek	Smolice	POL	2014	up now	yes
80	ni	Soldo	na	GER	2013	up now	yes
81	ni	Ella	na	FRA	2012	up now	yes
82	ni	Rezus	Smolice	POL	2018	up now	yes
83	ni	RGT Planet	na	FRA	2016	up now	yes

na—data not available; ni—not included in the gene bank collection; FRA—France; GER—Germany; POL—Poland.

### 4.2. DArTseq Genotyping

Seeds, were obtained from long term storage of NCPGR or directly from breeding stations, were sown in a greenhouse in a substrate dedicated to planting seeds. From eight, random seedlings in the second leaf stage, the middle part of the second leaf about 10 mm long was collected. A modified CTAB protocol [81,82] was used to isolate total genomic DNA. The DNA quantity and quality were assessed by spectrophotometric analysis using a NanoDrop ND-1000 spectrophotometer (NanoDrop Technologies, Willmington, DA, USA) followed by agarose gel electrophoresis (1.5% agarose). The obtained DNA isolates were mixed in equal proportions to form a pooled sample representing the tested cultivar. All bulk samples were diluted to a final concentration of 75 ng/µL and shipped to the Diversity Arrays Technology Pty Ltd., Canberra, Australia for DArTseq genotyping. The resulting sequences were aligned to the barley Morex genome assembly [21].

### 4.3. Data Analysis

DArTseq results in a form of a table containing codominant single nucleotide polymorphisms (SNPs) were transformed into a binary matrix. Each locus was recorded as two lines where homozygotes were denoted as 1/1 or 0/0 and heterozygotes as 1/0. In the first step the array was filtered according to reproducibility (RepAvg ≥ 0.95), call rate (CallRate ≥ 0.95), and the minor allele frequency (MAF > 0.01).

Further preliminary analysis included determination of the proportion of polymorphic loci and calculation of polymorphic information content (PIC), observed (Ho) and expected heterozygosity (He), and inbreeding coefficient (F) according to the formulas published in Dziurdziak et al. [25].

The distribution of the investigated loci on the chromosomes and PIC, Ho and F along the chromosomes were also assessed using the sliding window method with 500 kb intervals at 250 positions for each chromosome.

Values of variation coefficients were calculated for groups of cultivars using a formula excluding the effect of sample size. Analysis of variance ANOVA and Tukey’s post hoc test were used to compare the degree of variation. The level of allelic richness (AR) was assessed based on rarefaction method. Analysis of molecular variance AMOVA was also performed. The Wright’s FST parameter was used to estimate genome wide group differentiation, and to increase plot resolution transformation by rising FST to the 10th power (FST^10^) was performed [58].

The genetic distance between the sites was calculated using the Jaccard coefficient and then principal coordinate analysis (PCoA) was performed. Moreover, the identity by descent (IBD) was estimated for all pairwise comparisons among the accessions. Duplicates were defined as having IBD > 0.95 among accessions.

The final step of the analysis was to perform clustering based on the Bayesian model to analyze the genetic structure of examined accessions. In order to obtain the most probable value of K, a search was conducted in the range from 1 to 16 with six independent repetitions per K for cultivars analysis, whereas analysis of the compiled cultivars and preexisting landraces results was performed for K up to 11 with six independent runs/K. A LINUX cluster hosted by the Interdisciplinary Centre for Mathematical and Computational Modelling at the Warsaw University was used to run the analysis of batch files. The number of clusters was determined based on the posteriori data probability for a given K and ΔK [23] and the full search algorithm was used to find the best match for replicated cluster analysis results. A cutoff value of 0.8 was set as the probability of assigning accession to the group.

A core collection was extracted using the advanced M strategy implemented through a modified heuristic algorithm (A*).

The above mentioned analyses were performed using Microsoft Excel 2016, XLSTAT Ecology (Addinsoft, Inc., Brooklyn, NY, USA), GenAlEx 6.501 [22], HP-RARE 1.1 [83], PLINK [84], STRUCTURE v2.3.4 [85], CLUMPP [24], PowerCore [86]. The following packages in R were used to visualize the results: igraph [87], circlize [88]. The population structure analyses were performed in the framework of Computational Grant (G72-19) from the Interdisciplinary Center for Mathematical and Computer Modeling at the University of Warsaw, Poland (ICM UW).

## 5. Conclusions

This study showed that the gene pool structure of spring barley cultivars has changed significantly during 120 years of breeding in Poland. Many alleles have been displaced and replaced by new ones. These changes were associated with breeding priority evolution over time. Traces of directed selection are particularly visible on chromosomes 3H and 5H. The genetic uniformity of the cultivars increased with the progress of breeding. In contrast, the low variation within some of the old cultivars is the result of selection that probably occurred before they were obtained by the gene bank. A side effect of the analysis was the identification and verification of duplicates and the establishment of a core collection and thus DArTseq analysis will contribute to more efficient management of the barley collection in the gene bank. Analysis of changes in the level of genetic diversity over time may not reflect changes in genetic structure, so its results should be treated with caution.

## Figures and Tables

**Table 1:** Summary of DArTseq loci distribution on chromosomes for 83 spring barley cultivars. Chromosome lengths according to barley Morex genome assembly [21].

Chromosome	Length (Mbp)	Number of Loci	Mean Distance (Mbp)	Percentage of Loci	Percentage of Homozygous Loci/Chromosome
1H	558.54	1150	0.49	10%	25%
2H	768.08	1695	0.45	15%	32%
3H	699.71	1541	0.45	13%	19%
4H	647.06	1109	0.58	10%	30%
5H	670.03	1574	0.43	14%	15%
6H	583.38	1158	0.50	10%	20%
7H	657.22	1571	0.42	13%	23%
Unknown	na	1857	na	16%	22%

**Table 2:** Summary of point mutation abundance at the studied loci by chromosome based on DArTseq analysis of 83 spring barley cultivars.

			Total	Abundance on Chromosomes							Unknown
				1H	2H	3H	4H	5H	6H	7H	
Transitions (Ts)	Purines	A > G	1910	186	263	235	206	278	183	245	314
		G > A	1752	178	254	211	168	249	193	228	271
	Pyrimidines	C > T	1754	184	248	252	165	240	169	243	253
		T > C	1604	160	222	244	145	207	151	237	238
Transversion (Tv)	Purines > Pyrimidines	A > C	522	33	70	75	47	70	56	70	101
		A > T	292	37	44	24	34	45	33	32	43
		G > C	890	101	119	107	90	116	80	125	152
		G > T	525	56	95	65	45	72	52	72	68
	Pyrimidines > Purines	C > A	533	45	79	67	44	72	47	91	88
		C > G	1027	89	170	146	86	118	103	128	187
		T > A	291	28	45	39	23	48	32	36	40
		T > G	555	53	86	76	56	59	59	64	102
% Ts			60.2%	61.6%	58.2%	61.1%	61.7%	61.9%	60.1%	60.7%	57.9%
% Tv			39.8%	38.4%	41.8%	38.9%	38.3%	38.1%	39.9%	39.3%	42.1%
Ts/Tv ratio			1.51	1.60	1.39	1.57	1.61	1.62	1.51	1.54	1.38

**Table 3:** Change in the number of unique alleles in consecutive breeding periods considering chromosome allocation. Results based on DArTseq analysis for 83 spring barley cultivars.

		Groups							
		Before 1945 vs. 1945–1969		1945–1969 vs. 1970–1989		1970–1989 vs. 1990–1999		1990–1999 vs. After 2000	
Chromosomes	1H	0	1	3	21	3	0	23	26
	2H	36	2	10	23	2	4	21	68
	3H	12	18	25	16	2	0	33	3
	4H	1	24	9	6	2	24	55	9
	5H	3	43	30	32	33	9	59	54
	6H	13	2	44	2	0	3	13	3
	7H	6	18	8	8	2	8	13	59
	Unknown	7	17	24	17	10	7	35	54

**Table 4:** Summary of Jaccard genetic distance analysis for 83 spring barley cultivars based on DArTseq-derived SNPs.

	Minimum		Maximum	
	Genetic Distance	Cultivars	Genetic Distance	Cultivars
Before 1945	0.162	Danubia Ciołkowski–Hanna Borzymowski	0.462	Cesarski Stieglera–Puławski Browarny
1945–1969	0.015	Jarek–Jarek	0.667	Kos–Mazowiecki
1970–1989	0.014	Klimek–Klimek	0.632	Klimek_(36)_–Polon
1990–1999	0.021	Rambo–Rambo	0.649	Start–Bryl_(59)_
after 2000	0.249	Granal–Sezam	0.450	Atico–Ella
total	0.014	Klimek–Klimek	0.677	Mazowiecki–Stratus

The number in parentheses is according to Table 6.

**Table 5:** List of duplicate accessions verified from passport data and DArTseq analysis results.

No.	Accession Number	Cultivar Name	Passport Data	Genetic Distance	Population Structure	Identity by Descent
17	PL42124	Damazy	yes	yes	yes	yes
18	PL43614	Damazy				
19	PL40940	Jarek	yes	yes	yes	yes
20	PL42363	Jarek				
28	PL41419	Ars	yes	no	yes	yes
29	PL43646	Ars				
41	PL41418	Mago	no			
30	PL43423	Bielik	yes	yes	yes	yes
31	PL41415	Bielik				
32	PL43033	Dema	yes	no	no	no
33	PL43416	Dema				
35	PL43086	Klimek	yes	yes	yes	yes
36	PL43414	Klimek				
38	PL43032	Lot	yes	no	no	no
39	PL43421	Lot				
44	PL43056	Rudzik	yes	yes	yes	yes
45	PL43423	Rudzik				
51	PL43335	Orlik	yes	yes	yes	yes
52	PL43417	Orlik				
53	PL43368	Polo	yes	no	no	no
54	PL43411	Polo				
59	PL500074	Bryl	yes	yes	yes	yes
60	PL43949	Bryl				
61	PL43812	Bryl		no	no	no
62	PL500070	Edgar	yes	yes	yes	yes
63	PL500666	Edgar				
64	PL43419	Nagrad	yes	yes	yes	yes
65	PL43379	Nagrad				
67	PL500667	Rambo	yes	yes	yes	yes
68	PL43747	Rambo				
70	PL43369	Rodos	yes	no	no	no
71	PL43412	Rodos				

## Data Availability

The raw data of DArTseq SNP used in this study are openly available on the platform Center for Open Science at https://osf.io/v4m5s/ (accessed on 17 April 2022).

## Supplementary Materials

The following supporting information can be downloaded at: https://www.mdpi.com/article/10.3390/ijms23094553/s1.
