# Supplementary figures and images for: Tracking Changes in the Spring Barley Gene Pool in Poland during 120 Years of Breeding
Source: Int J Mol Sci. 2022 Apr 20;23(9):4553. doi: 10.3390/ijms23094553 (PMC9099733; doi:10.3390/ijms23094553)

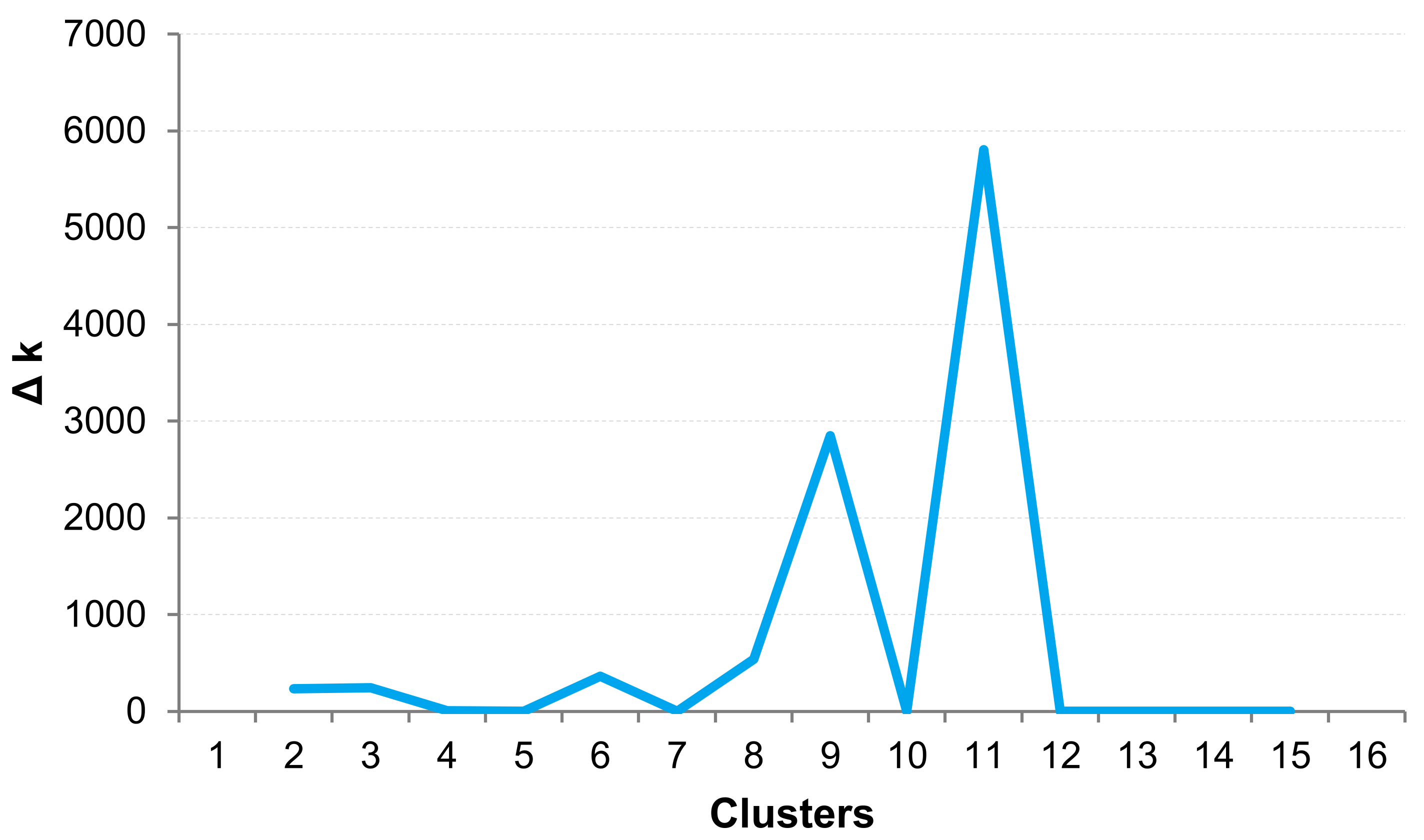

Supplement: Supplementary file 1 [file ijms-23-04553-s001.zip › Supplementary Files/Figure S2.tiff]
